# Parastomal hernia prevention with permanent mesh in end colostomy: failure with late follow-up of cohorts in three randomized trials

**DOI:** 10.1007/s10029-023-02781-4

**Published:** 2023-03-25

**Authors:** M. López-Cano, M. Adell-Trapé, M. Verdaguer-Tremolosa, V. Rodrigues-Gonçalves, J. Badia-Closa, X. Serra-Aracil

**Affiliations:** 1 Abdominal Wall Unit, Department of General Surgery, Hospital Universitari Vall d’Hebron, Universitat Autònoma de Barcelona, Barcelona, Spain; 2 Department of General and Digestive Surgery, Colorectal Unit, Parc Taulí University Hospital, Universitat Autònoma de Barcelona, Sabadell, Barcelona, Spain; 3 Abdominal Wall Unit, Department of General Surgery, Hospital Universitari Vall d’Hebron, Passeig Vall d’Hebron 119-129, 08035 Barcelona, Spain

**Keywords:** Parastomal, Hernia, Prevention, Mesh

## Abstract

**Purpose:**

Short-term results have been reported regarding parastomal hernia (PH) prevention with a permanent mesh. Long-term results are scarce. The objective was to assess the long-term PH occurrence after a prophylactic synthetic non-absorbable mesh.

**Methods:**

Long-term data of three randomized controlled trials (RCTs) were collected. The primary outcome was the detection of PH based exclusively on a radiological diagnosis by computed tomography (CT) performed during the long-term follow-up. The Kaplan–Meier method was used for the comparison of time to diagnosis of PH according to the presence of mesh vs. no-mesh and the technique of mesh insertion: open retromuscular, laparoscopic keyhole, and laparoscopic modified Sugarbaker.

**Results:**

We studied 121 patients (87 men, median age 70 years), 82 (67.8%) of which developed a PH. The median overall length of follow-up was 48.5 months [interquartile range (IQR) 14.4–104.9], with a median time until PH diagnosis of 17.7 months (IQR 9.3–49.0). The survival analysis did not show significant differences in the time to development of a PH according to the presence or absence of a prophylactic mesh neither in the overall study population (log-rank, *P* = 0.094) nor in the groups of each technique of mesh insertion, although according to the surgical technique, a higher reduction in the appearance of PH for the open retromuscular technique was found (log-rank, *P* = 0.001).

**Conclusion:**

In the long-term follow-up placement of a non-absorbable synthetic prophylactic mesh in the context of an elective end colostomy does not seem effective for preventing PH.

## Introduction

Development of parastomal hernia (PH) after intestinal stoma creation continues to be a matter of concern due to its high prevalence [1], marked impact on the patient’s quality of life [2], and poor results associated with surgical hernia repair both in the short term [3] and long term [4]. There has been a renewed interest especially in the last decade, to establish preventive methods of PH formation, in particular using a prophylactic non-absorbable synthetic mesh during end colostomy formation in elective surgical contexts [5]. In this setting, the results of systematic reviews and meta-analyses of randomized controlled trials (RCTs) of diverse methodological quality have consistently shown the usefulness of this type of mesh in the prevention of PH [6]. Moreover, benefits of prophylactic mesh placement have been supported by the recommendation of the European Hernia Society (EHS) published in 2018 for the use of prophylactic mesh when an elective permanent end colostomy is constructed [7]. However, between 2019 and 2022, there has been an important controversy based on negative findings of three RCTs [8–10] and one meta-analysis [11] as opposed to positive results of an updated systematic review and meta-analysis of all previous RCTs published in the literature [12]. In review, it was concluded that there remains a significant reduction in the risk of PH with the use of prophylactic mesh at the time of end colostomy formation [12].

It is possible that an important drawback in the interpretation of the past and recent evidence may be related to the short follow-up of RCTs published so far [11, 12], with a prolonged follow-up in only four studies [13–16]. On the other hand, a further difficulty in the interpretation of findings is the use of different surgical techniques for mesh placement, with the most frequent being a retromuscular position using an open approach (laparotomy) or an intraperitoneal position using a laparoscopic approach (keyhole or modified Sugarbaker) [5, 12].

In the previous context, the main objective of this study was to assess the long-term effectiveness of the use of prophylactic mesh at the time of elective end colostomy formation based on a pooled analysis of three RCTs [17–19], published by two different groups of the same geographical area and using three different procedures: open retromuscular [17], laparoscopic keyhole [18], and laparoscopic modified Sugarbaker [19].

## Methods

Between 2009 and 2016, three RCTs carried out by two independent collaborative groups in acute-care tertiary hospitals located in the same geographical area, reported short-term outcomes (maximum 2 years) of the use of a permanent prophylactic synthetic mesh for preventing PH in patients with definitive end colostomy. Two studies were single-center trials [17, 18] and the third study was a multicenter trial [19]. In all three trials, patients were randomly assigned with a 1:1 allocation ratio to mesh placement (intervention group) versus no-mesh (control group) at the time of elective surgery. The study design, the surgical procedure, and approval by the institutional review board of the three trials were reported in the corresponding publications of short-term results. Patients included in the three trials were initially informed of the possibility of participating in a retrospective study aimed to collect long-term follow-up data.

Briefly, and by chronological order, salient features of these three RCTs are here described:

- The first RCT [17] analyzed the prevalence of PH after colostomy placement. Fifty-four patients were included, 27 allocated to the intervention (mesh) and 27 to the control group. Patients were scheduled for permanent end colostomy surgery to treat cancer of the lower third of the rectum. A permanent synthetic lightweight mesh was inserted in the retromuscular position (open approach) in the intervention group. Computed tomography (CT) evaluation of the presence of PH revealed 14/27 (44.4%) hernias in the control group and 6/27 (22.2%) in the mesh group (*P* = 0.083), whereas by clinical assessment, 11/27 (40.7%) PH were diagnosed in the control group and 4/27 (14.8%) in the mesh group (*P* = 0.033).
- In the second RCT [18], 36 patients were randomized, 19 to the mesh group and 17 to the control group. The study population included rectal cancer patients undergoing elective laparoscopic abdominoperineal resection with permanent colostomy. A large-pore lightweight composite mesh in the intraperitoneal/onlay position was inserted by laparoscopic approach in patients randomized to the intervention group. The mesh had a cruciate incision on the center to allow the colon passage through that opening (keyhole). PH was defined radiologically by a CT scan performed 12 months after surgery. PH detected in 50% of patients in the mesh group and in 93.8% of patients in the control group (*P* = 0.008).
- In the third RCT [19] rectal cancer patients undergoing laparoscopic abdominoperineal resection with permanent colostomy were randomized to the mesh group (*n* = 24) or to the control group (*n* = 28). In the intervention group, a prophylactic permanent synthetic composite lightweight mesh, without incisions on its surface, was placed intraperitoneally using a laparoscopic approach. The mesh was positioned on the lateralized colon covering the stoma orifice. In this way a tunnel between the abdominal wall and the prosthesis was created (modified Sugarbaker technique). After CT examination, 6/24 PHs (25%) were observed in the mesh group compared with 18/28 (64.3%) in the non-mesh group (*P* < 0.005).

The present study included all patients from the aforementioned three RCTs. The primary outcome was the detection of PH based exclusively on a radiological diagnosis by CT examination performed over the long-term follow-up of patients. CT scans were performed in the supine position, with the patient at rest, and with intravenous contrast. Because no consistent radiological criteria for PH have been used in different studies [17–19], we here considered a wide definition of PH to describe a loop of intestine or any abdominal organ, as well as preperitoneal fat, protruding through the defect alongside the ostomy. All CTs were evaluated by a single experienced radiologist who was unaware of the surgical technique used for mesh placement.

The start of follow-up was considered as the date of CT examination from which the short-term data were extracted in the original studies [17–19], and the end of follow-up (whether the patient was alive or death) as the date of the last CT scan performed. All patients lost to long-term follow-up for any reason were excluded. The patients were not clinically evaluated given the interobserver variation of the clinical diagnosis in the case of PH [20], considering the CT scan as the most objective procedure for the purpose of this study [21]. The secondary outcome of the study was the number of patients with PH requiring surgical hernia repair.

### Statistical analysis

Categorical variables are expressed as frequencies and percentages, and continuous variables as mean and standard deviation (SD) or median and interquartile range (IQR) (25th–75th percentiles) as appropriate. Differences in the distribution of variables (demographic data, body mass index [BMI], occurrence of PH, length of follow-up, and mortality) between patients in the mesh and non-mesh (control) groups, as well as according to the surgical technique of mesh insertion, were analyzed with the chi-square test or the Fisher’s exact test for categorical data, and the Mann–Whitney *U* test for continuous data. The Kaplan–Meier method was used for the comparison of survival times (time to diagnosis of PH) according to the presence of mesh and the surgical technique used for mesh insertion. Differences in survival were analyzed with the log-rank test. Statistical significance was set at *P* < 0.05. The SPSS version 26.0 (IBM Corp., Armonk, NY, USA) was used for data analysis.

## Results

Of a total of 184 patients included in the three RCTs, 63 were excluded from the analysis due to insufficient data in 44 and lost to follow-up in 19.Therefore, the study population consisted of 121 patients (65.8%), 87 men and 34 women, with a median age of 70 years (IQR 61–76 years) and BMI of 26.3 kg/m^2^ (IQR 24.2–28.6 kg/m^2^). Eighty-two patients developed a PH (30 in the mesh group and 52 in the non-mesh group), with a total rate of PH of 67.8%. The median overall length of follow-up was 48.5 months (IQR 14.4–104.9 months), with a median time until diagnosis of PH of 17.7 months (IQR 9.3–49.0 months).

The distribution of variables according to the surgical technique of mesh insertion is shown in Table 1. The percentage of male patients was significantly higher in the modified Sugarbaker group as compared to the other groups (*P* = 0.008) and the rate of PH was significantly higher in the keyhole group (*P* = 0.008). Also, the length of follow-up was significantly longer in the keyhole group (*P* = 0.003), but time to appearance of PH was significantly shorter in this group (*P* = 0.011). The mortality rate was also higher in the keyhole group (*P* = 0.049). The comparison of variables according to the presence of mesh vs. non-mesh in the three surgical techniques of mesh insertion is shown in Table 2. In the mesh group, 100% of patients undergoing mesh insertion using the keyhole technique developed a PH as compared to 55.6% in the modified Sugarbaker group and 45% in the retromuscular group (*P* = 0.005). The keyhole group showed a higher length of overall follow-up (*P* = 0.001) and a shorter time until development of PH (*P* = 0.089). The mortality rate was also significantly higher among non-mesh controls of the keyhole group (*P* = 0.027).

**Table 1:** Variables distribution according to the surgical technique of mesh insertion

Study group (*N*:121)	Modified sugarbaker (*N*:44)	Keyhole (*N*:33)	Retromuscular (*N*:44)	*P *value
Mesh [yes (%)]	18 (40.9)	11 (33.3)	20 (45.5)	0.561
Woman (%)/men (%)	8 (18.2)/36 (81.8)	16 (48.5)/17 (51.5)	10 (22.7)/34 (77.3)	0.008
Age years, median(IQR)	72 (60–78)	72 (66–75)	67 (61–73)	0.280
BMI, median(IQR)	26.2 (24.2–28.0)	26.9 (24.7–29.0)	26.6 (24.2–29.0)	0.540
PH, *N* (%)	29 (65.9)	29 (87.9)	24 (54.5)	0.008
Months to PH, median(IQR)	17.9 (9.4–41.8)	14.5 (8.1–20.7)	24.7 (11.3–92.4)	0.011
Exitus, *N* (%)	19 (43.2)	18 (54.5)	12 (27.3)	0.049
Months of follow- up, median(IQR)	30.1 (12.9–70.3)	104.2 (32.3–140.1)	42.2 (11.3–138.8)	0.003

*P* probability, *IQR* interquartile range, *BMI* body mass index, *PH* parastomal hernia

**Table 2:** Variables comparison according to the presence mesh vs. non-mesh in the three surgical techniques

Study group	121 Patients							
Subgroup	Mesh (*n*:49)			*P*	No mesh (*n*:72)			*P*
Type of technique	Mod Sugarbaker	Keyhole	Retromuscular		Mod Sugarbaker	Keyhole	Retromuscular	
*N* (%)	18 (37)	11 (22)	20 (41)		26 (36)	22 (31)	24 (33)	
Age years, median (IQR)	74 (60.7–78.5)	72 (66–75)	69.5 (60.2–73.0)	0.343	70 (58.2–78.0)	71.5 (66.2–77.5)	64.5 (62–74.7)	0.501
BMI, median (IQR)	26 (24.5–27.9)	25.8 (23.3–27.3)	26.1 (24.2–28.4)	0.782	26.3 (22.4–28.3)	27.6 (25.3–29.2)	27.3 (24.2–29.3)	0.411
PH, *N* (%)	10 (55.6)	11 (100.0)	9 (45.0)	0.005	19 (73.1)	18 (81.8)	15 (62.5)	0.341
Months to PH, median (IQR)	20.5 (15.9–60.5)	13.3 (6.6–30.2)	50.4 (11.6–88.1)	0.089	12.7 (8.6–31.1)	14.8 (8.5–20.3)	22 (11.1–98.1)	0.110
Exitus, *N* (%)	9 (50.0)	4 (36.4)	6 (30.0)	0.491	10 (38.5)	14 (63.6)	6 (25.0)	0.027
Months of follow- up, median (IQR)	35.2 (18.4–63.4)	129.8 (101.1–151)	59.1 (14.4–155.0)	0.001	28.3 (12.3–74.4)	63 (18.9–119.3)	23.6 (11.1–100.8)	0.174

*P* probability, *BMI* bodymassindex, *PH* parastomalhernia, *IQR* interquartilerange

**Fig. 1:**
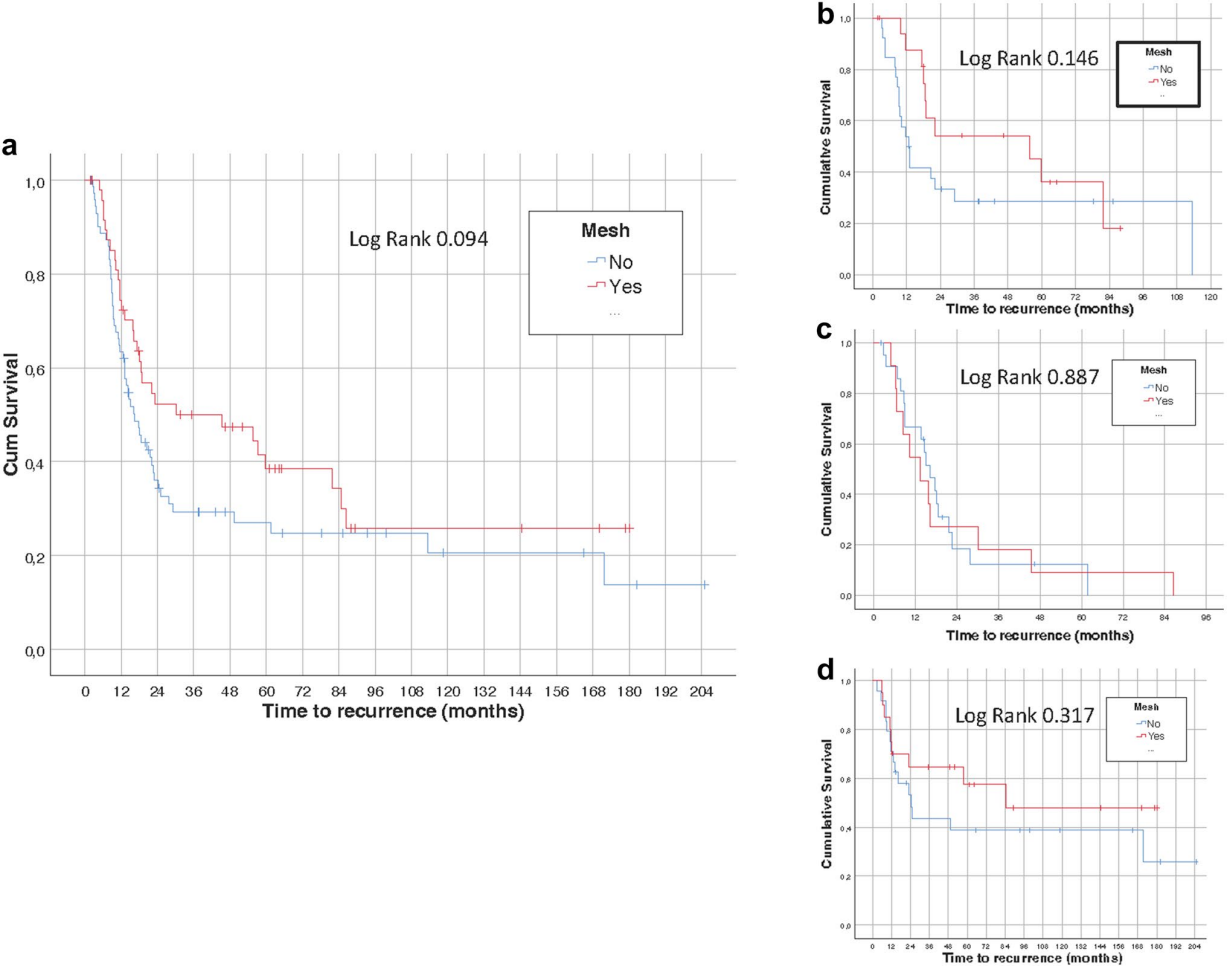
Time-to-event analysis according end colostomy formation with and without the use of a prophylactic synthetic mesh: **a** overall study population, **b** laparoscopic modified Sugarbaker, **c** laparoscopic keyhole, **d** open retromuscular

**Fig. 2:**
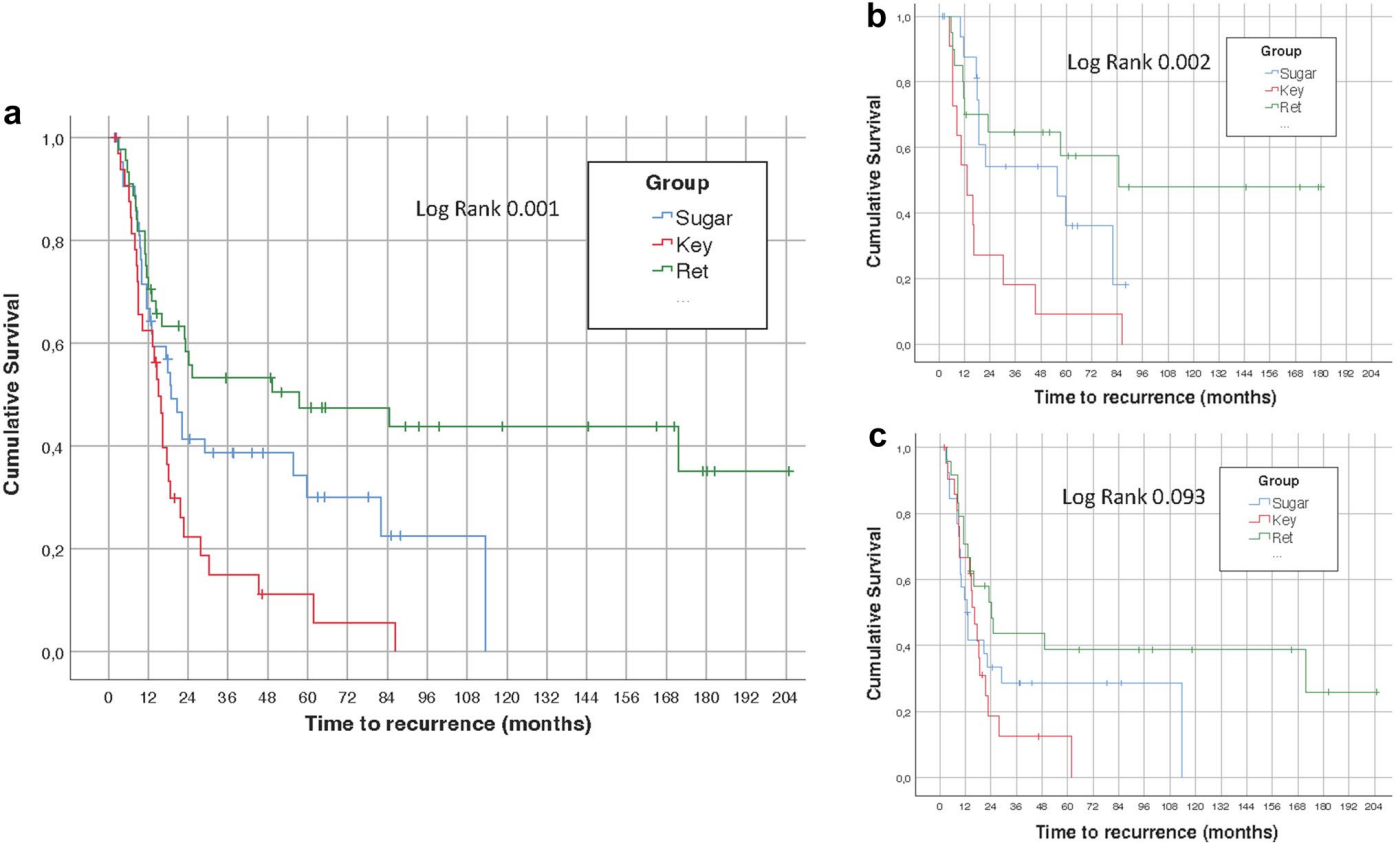
Time-to-event analysis according to the surgical technique of mesh placement: **a** overall study population, **b** with mesh, **c** without mesh

The survival analysis did not show significant differences in the time to development of a PH according to the presence or absence of a prophylactic mesh, neither in the overall study population (log-rank, *P* = 0.094) (Fig. 1a) nor in the groups of each mesh insertion technique (Fig. 1b, c, d). Figure 2 shows the results of survival analysis according to the presence or absence of mesh and the surgical procedures in the overall study population (Fig. 2a), with a higher reduction in the appearance of PH for the retromuscular technique (log-rank, *P* = 0.001) (Fig. 2b). Differences among surgical procedures in the group without mesh were not statistically significant (Fig. 2c).

Among patients with PH, hernia repair was carried out in: 4 (1 mesh and 3 non-mesh) of the 29 patients in the modified Sugarbaker group (13.8%); 10 (4 mesh and 6 non-mesh) of the 29 patients in the keyhole technique (34.5%) and 5 (1 mesh and 4 non-mesh) of the 24 patients in the retromuscular technique (20.8%).

## Discussion

The present analysis of the efficacy of a synthetic mesh in the prevention of PH showed that mesh placement at the time of end colostomy construction was not associated with a preventive effect in the long term, neither for the laparoscopic approach using a modified Sugarbaker technique or an intraperitoneal keyhole procedure, not for an open approach using a retromuscular insertion technique. A total rate of PH of 67.8% is very discouraging. However, despite the lack of significant overall efficacy, the retromuscular open technique showed the most favorable results.

It should be noted that the overwhelming majority of information in the literature about the prevention of PH with mesh refers to the use of permanent synthetic mesh in patients undergoing permanent end colostomy in an elective surgery setting [5, 6, 11, 12] and that all comments in this discussion will refer preventing the development of PH formation in this context. We are aware that the global evidence in favor of the use of a prophylactic mesh for the prevention of PH is not uniform regarding quality and risk of bias (methodology, mesh placement technique, definition and diagnosis of PH), and that surgeons cannot be equally confident in the results of all systematic reviews and meta-analyses published on this topic [6]. However, recent publications of meta-analysis against [11] and in favor [12] of the efficacy of prophylactic mesh for PH prevention generates confusion in clinical decision making. In our opinion, one of the main concerns is the lack of long-term results. Short-term results (maximum at 1 or 2 years) are reported by all of these meta-analyses. In our study, the overall median length of follow-up until development of PH was 17.7 months (IQR 9.3–49.0), which may indicate that probably a short-term follow-up may not be the most appropriate time frame to assess the occurrence of PH after a prophylactic mesh. In addition, the surgical procedures for mesh placement reported in the literature are heterogeneous, with the retromuscular technique and an open approach being the most commonly used [5, 6, 11, 12].

We agree with other authors that perhaps not all clinical research on the prevention of PH with a mesh should be limited to whether or not PH occurs [22]; probably it may be necessary to investigate other aspects related to PH as a pathological condition far beyond its presence or absence. Some aspects, such the impact of the various PH subtypes on the quality of life, the patient’s characteristics, or the different types of stomas associated with PH may be considered before performing any prophylactic intervention. Other aspects after the intervention may be considered, such as the influence of the type of mesh and its position, PH repair rates, or the importance of hernia repair in the context of a previous prophylactic mesh. However, these interesting quantitative and qualitative characteristics need to be evaluated in futures studies [22]. In our opinion, recommendation of a non-absorbable synthetic prophylactic mesh in a patient requiring a permanent end colostomy should be currently based on the consideration of the likelihood of development of PH in the individual patient and the practice of a shared decision-making process. This process entails a relationship of trust between patient and surgeon, where the surgeon must recognize and respect the autonomy of the patient and the patient must have reasonable knowledge of the problems related to the prevention of PH and the risk and benefits of the different surgical options [23, 24].

Evidence of previous RCTs together with long-term data provided by the analysis of the present study may be useful during the shared decision-making process when a prophylactic mesh is proposed for preventing a PH. In this respect, we believe that the patient can be informed according to the following considerations: (a) data in the literature refer only to the risk of developing PH; (b) the final impact of the mesh on the patient’s quality of life remains unclear whether in the presence or absence of PH, as well as the most appropriate mesh and its position, or which is the most appropriate patient to place the prophylactic mesh; (c) in the short term (< 2 years), a mesh seems to be effective using laparoscopic techniques, although there are few studies using two different techniques (keyhole and modified Sugarbaker) and with a small sample size [5, 12]; (d) in the short term (< 2 years), the efficacy of a mesh using open retromuscular techniques is controversial [5, 11, 12]; (e) in the long term (3 or more years), previous evidence of ineffectiveness of a mesh when a laparoscopic keyhole technique is used [15] or an open retromuscular technique is used [16] were also confirmed in our analysis, which also showed ineffectiveness of the modified Sugarbaker technique; and (f) in the long term (3 years or more), a mesh seems effective in two studies in which an open retromuscular technique was used [13, 14]. According to the present findings, the use of a mesh in this context does not appear to be effective, although the retromuscular position would be the most appropriate for the prevention of PH as compared with the laparoscopic techniques.

In these circumstances, it is very difficult to solve the question of whether a prophylactic mesh should be used or not. It may be argued that it is finally a question of palliative prevention in terms of developing HP or not, and in this respect, it is interesting to consider the parallelism between PH prevention and treatment. In the latter, data from the literature in different registries (real-world evidence) show a very high recurrence rate in the short term [3] and high probabilities of recurrence in the long term [25]. Both treatment and prevention of PH may be palliative in the exclusive terms of developing PH or not.

Regarding the number of PH repairs following preventing attempts, it has been reported that placement of a prophylactic mesh reduces significantly the rates of hernia repairs and is associated with a decrease of clinically significant PH rates [15, 22]. In the present analysis, the majority of repaired PH was also in the non-mesh group. However, the strength of these data is low and more studies will be needed to clarify this “protective effect” of the mesh. Among other reasons, surgeons may be reluctant to perform reoperations in patients with a previous mesh due to potential technical difficulties. Also, registry data seem to indicate that the cumulative incidence of recurrent PH repair at 5 years may be as low as 5% (95% CI 3% to 5%) [25]. Data of our study showed variable rates of PH repair after prevention attempt with percentages of 13.8%, 20.8%, and 34.5% according to the technique used, but quite consistent with results reported in the aforementioned registries, where the cumulative incidence of a primary repair was 9% (95% CI 8% to 11%) within 1 year and 19% (95% CI 17% to 22%) within 5 years after the occurrence of a PH [25]. Therefore, it is probable that more robust evidence is needed to confirm the “protective effect” of a prophylactic mesh, particularly as previously mentioned, taking into account how little is known of the impact of mesh on the patient's quality of life and what occurs when a PH develops in a patient with a previous prophylactic mesh.

Limitations of the study are the relatively small number of patients and the intrinsic heterogeneity of the analyses mainly the difference in mesh placement approach, however, all the patients presented the same underlying pathology, with the same prevention procedure (i.e. non-absorbable synthetic prophylactic mesh) and identical endpoints after an analysis of randomized parallel groups. Also, no patient was clinically examined. Other characteristics, such as the effect of mesh vs. non-mesh on the quality of life or PH repair in the context of a previous prophylactic mesh were not evaluated. Strengths of the study include the fact that radiological diagnosis of PH was blinded for the different study groups.

In summary, the effectiveness of a non-absorbable prophylactic mesh in the context of an elective permanent end colostomy for preventing PH in the long term remains unproven. Even so the technique that performed best in terms of prevention was the retromuscular procedure and the worst the keyhole technique. The “protective effect” of a prophylactic mesh in terms of reducing the rates of interventions or clinically significant PH remains to be elucidated. Placement of a mesh for the prevention of PH in terms of appearance vs. non-appearance of a PH shows that the incidences of PH are high and perhaps going even higher with longer follow-up. In the previous context, PH prevention with permanent mesh in end colostomy is at best considered a delaying strategy.

## Data Availability

The study data are available from the corresponding author upon request.
